# Multi-omics data integration for hepatocellular carcinoma subtyping with multi-kernel learning

**DOI:** 10.3389/fgene.2022.962870

**Published:** 2022-09-06

**Authors:** Jiaying Wang, Yuting Miao, Lingmei Li, Yongqing Wu, Yan Ren, Yuehua Cui, Hongyan Cao

**Affiliations:** ^1^ Department of Respiratory, Gastroenterology and Oncology (West Branch), The Second Hospital of Shanxi Medical University, Taiyuan, China; ^2^ Division of Health Statistics, School of Public Health, Shanxi Medical University, Taiyuan, China; ^3^ Department of Psychiatry, Third Hospital of Shanxi Medical University, Shanxi Bethune Hospital, Shanxi Academy of Medical Sciences, Tongji Shanxi Hospital, Taiyuan, China; ^4^ Tongji Hospital, Tongji Medical College, Huazhong University of Science and Technology, Wuhan, China; ^5^ Department of Statistics and Probability, Michigan State University, East Lansing, MI, United States; ^6^ Shanxi Medical University-Yidu Cloud Institute of Medical Data Science, Taiyuan, China

**Keywords:** biomarkers, omics data integration, rMKL-LPP, subtype identification, multiple kernel learning

## Abstract

Hepatocellular carcinoma (HCC) is a leading malignant liver tumor with high mortality and morbidity. Patients at the same stage can be defined as different molecular subtypes associated with specific genomic disorders and clinical features. Thus, identifying subtypes is essential to realize efficient treatment and improve survival outcomes of HCC patients. Here, we applied a regularized multiple kernel learning with locality preserving projections method to integrate mRNA, miRNA and DNA methylation data of HCC patients to identify subtypes. We identified two HCC subtypes significantly correlated with the overall survival. The patient 3-years mortality rates in the high-risk and low-risk group was 51.0% and 23.5%, respectively. The high-risk group HCC patients were 3.37 times higher in death risk compared to the low-risk group after adjusting for clinically relevant covariates. A total of 196 differentially expressed mRNAs, 2,151 differentially methylated genes and 58 differentially expressed miRNAs were identified between the two subtypes. Additionally, pathway activity analysis showed that the activities of six pathways between the two subtypes were significantly different. Immune cell infiltration analysis revealed that the abundance of nine immune cells differed significantly between the two subtypes. We further applied the weighted gene co-expression network analysis to identify gene modules that may affect patients prognosis. Among the identified modules, the key module genes significantly associated with prognosis were found to be involved in multiple biological processes and pathways, revealing the mechanism underlying the progression of HCC. Hub gene analysis showed that the expression levels of *CDK1*, *CDCA8*, *TACC3*, and *NCAPG* were significantly associated with HCC prognosis. Our findings may bring novel insights into the subtypes of HCC and promote the realization of precision medicine.

## 1 Introduction

Hepatocellular carcinoma (HCC), a primary malignant neoplasm, accounts for approximately 90% of cases of all liver cancers (Licata et al., 2021). It has been reported to be the fastest growing cause of cancer-related death in the United States, and is expected to be the third leading cause of cancer-related death by 2030, if the trends remain (Rahib et al., 2014). The current therapy and management of HCC is based on the expected returns of the main interventions and tumor grades following the Barcelona Clinic Liver Cancer (BCLC) staging system (European Association for the Study of the Liver, 2018; Llovet et al., 2021). So far, the prognosis of HCC patients remains poor (Forner et al., 2018). Actually, patients at the same stage can be defined as different molecular subtypes according to major molecular drivers and pathways involved (Villanueva, 2019). Several works have been done to identify the HCC subtypes using mRNA gene expression data (Boyault et al., 2007; Hoshida et al., 2009; Goossens et al., 2015). However, any individual omics data can only reveal the intrinsic molecular characteristics of a tumor marginally. High throughput technology has enabled the acquisition of multi-omics data more easily. The joint analysis of multi-omics data types is being increasingly emphasized. Multi-omics data integrative analysis can offer insights into the crucial links between different types of omics data and further provide a thorough comprehension of the potential biological processes (Lock et al., 2013). Three HCC molecular subtypes were identified from 183 TCGA samples by integrating five data sources (DNA copy number, DNA methylation, mRNA expression, miRNA expression and RPPA) (Cancer Genome Atlas Research Network, 2017). These molecular subtypes associated with specific genomic disorders and clinical features, allow researchers to discover targets used as drug design and biomarkers for predicting response. However, the high heterogeneity and complex etiologic factors of HCC make the prognosis prediction very challenging (Colagrande et al., 2016). HCC still has a relatively high incidence of recurrence and low 5-year survival rate. Therefore, identifying accurate molecular subtypes and biomarkers is essential for developing new effective therapies to improve the prognosis of HCC patients.

Some multi-omics integrative clustering methods have been proposed using multiple data types for subtyping. They can be divided into four categories (Rappoport and Shamir, 2018): 1) Early integration methods that input a single large dataset obtained by merging multi-omics data for clustering; 2) Late integration methods that apply a two-step clustering method which first clusters each omics data and then integrates them; 3) Methods applying statistical modeling that assume a particular data distribution (sensitive to feature selections); and 4) Similarity-based methods (e.g. regularized Multiple Kernel Learning with Locality Preserving Projections, or rMKL-LPP) (Speicher and Pfeifer, 2015) that first create similarity matrices based on each data type, then integrate them for clustering. An advantage of these methods is that they allow incorporating diverse omics data types, such as categorical and ordinal data. rMKL-LPP was extended from a multiple kernel learning based dimensionality reduction method. Based on the input data, it conducts dimension reduction such that similarities between samples and their nearest neighbors are remained in a low dimensional space. rMKL-LPP uses multiple kernel matrices to preserve the degrees of similarity within each omics data. Considering the differences of matrices, rMKL-LPP upweight the matrices with high information content and assign low weights to those with low information content. Moreover, it added a regularization term in the optimization problem to avoid overfitting. Rappoport et al. (Rappoport and Shamir, 2018) provided a comprehensive comparison of different multi-omics clustering algorithms spanning ten different cancer types and pointed out that rMKL-LPP has superior performance in terms of clinical enrichment.

Epigenetic dysregulation such as modifications in DNA methylation or changes in levels of microRNAs, plays a critical role in HCC (Rebouissou and Nault, 2020). Gene expression analysis also revealed differentiation patterns among HCC (Hoshida et al., 2009). In this work, we proposed to use rMKL-LPP method to integrate DNA methylation, miRNA and mRNA expression data to obtain subtypes of HCC. Focusing on the subtypes of HCC, downstream analyses were performed to explore the molecular features and pathways with potential prognostic value to prolong patient survival time and further promote the realization of precision diagnosis and treatment.

## 2 Materials and methods

### 2.1 Data sources

We downloaded mRNA expression data, miRNA expression data, DNA methylation data and clinical data from The Cancer Genome Atlas (TCGA) for HCC using TCGAbiolinks R package (Zhou et al., 2015). The DNA methylation data were measured using Illumina Human Methylation 450 Beadchip.

### 2.2 Data preprocessing

We performed the same data preprocessing steps for the mRNA and miRNA expression data. Features with more than 30% missing rate were removed. The rest of the missing data were imputed applying K-nearest neighbor (KNN) method (Troyanskaya et al., 2001), followed by $log_{2}\left(x+1\right)$ transformation. For DNA methylation, we focused on the CpG sites in the promoter region. The promoter region is defined as the region within 2kb of a transcription start site (Gusev et al., 2014). CpG sites on sex chromosomes were excluded for further analysis. Subsequently, we removed features with more than 30% missing rate, and imputed the rest of missing data by applying a KNN method. Finally, 16,534 mRNA expression probes, 437 miRNAs, and 49,022 DNA methylation sites were obtained for 287 patients.

### 2.3 Statistical method

#### 2.3.1 rMKL-LPP

rMKL-LPP proposed by Speicher and Pfeifer (2015), is an extension of MKL-DR method that can perform dimensionality reduction and data integration simultaneously. To make the work complete, we briefly introduce the algorithm here.

##### 2.3.1.1 Multiple kernel learning

Given *M* datasets ${\left(x_{i}^{m}\right)}_{i=1,\cdots ,N}$ (for m=1,⋯,M ), all observed on the same samples i=1,⋯,N . *M* different kernels $K_{m}$ provided different views of the datasets, each related to different data type. Multi-kernel learning linearly combines multiple kernel matrices $\left\{K_{1},\cdots K_{M}\right\}$ into a composite kernel matrix *K*, i.e.,

$$K=\overset{M}{\underset{m=1}{\sum }}\beta_{m}K_{m},subject\;to\;\overset{M}{\underset{m=1}{\sum }}\beta_{m}=1,\beta_{m}\geq 0$$ (1)

where $\beta_{m}\prime s$ are the weight coefficients.

##### 2.3.1.2 Dimensionality reduction and parameter optimization

For a given set of input kernel matrices, we use the dimension reduction of Locality Preserving Projections (LPP) method (Rong et al., 2017) to maintain similarities between each sample and its nearest neighbors in a low dimensional space. The projection vector *v* is optimized according to the graph-preserving criterion:

$$\begin{array}{l} \min_{\upsilon }\overset{N}{\underset{i,j=1}{\sum }}{\left\|\upsilon^{T}x_{i}-\upsilon^{T}x_{j}\right\|}^{2}w_{ij}\\ subject\;to\;\overset{N}{\underset{i=1}{\sum }}{\left\|\upsilon^{T}x_{i}\right\|}^{2}d_{ij}=const.\\ \omega_{ij}=\left\{ \begin{array}{cc} 1, & \mathrm{if}\;i\in N_{k}\left(j\right),\vee j\in N_{k}\left(i\right)\\ 0, & \mathrm{otherwise} \end{array}\right.\\ d_{ij}=\left\{ \begin{array}{cc} \overset{N}{\underset{n=1}{\sum }}w_{in}, & if\;i=j\\ 0, & \mathrm{otherwise}. \end{array}\right. \end{array}$$ (2)

where $x_{i},x_{j}$ represents sample *i* and *j*, respectively; the elements $w_{ij}$ constitute the similarity matrix *W*; the elements $d_{ij}$ constitute the constraint matrix *D*; and $N_{k}\left(i\right)$ represents the k nearest neighbors of sample *i*. We chose 9 as the number of nearest neighbors for all datasets following Speicher and Pfeifer (2015).

The constrained optimization problem in (2) can be achieved by an implicit mapping of the features to a high-dimensional Hilbert space $\phi :x_{i}\to \left(x_{i}\right)$ . It can be demonstrated that the optimal projection vector *v* lies in the span of $x_{i}$ such that $v=\Sigma_{i=1}^{N}\alpha_{i}\text{ϕ}\left(x_{i}\right)$ (Rong et al., 2017). Based on the kernel function $K\left(x,x^{\prime }\right)=\langle \text{ϕ}\left(x_{i}\right),\text{ϕ}\left(x^{\prime }\right)\rangle$ and Eq. 1, adding the constraint on β , the following optimization problem is given:

$$\begin{array}{l} \min_{\alpha ,\beta }\overset{N}{\underset{i,j=1}{\sum }}{\left\|\alpha^{T}\kappa^{i}\beta -\alpha^{T}\kappa^{j}\beta \right\|}^{2}w_{ij}\\ subject\;to\;\sum_{i,j=1}^{N}{\left\|\alpha^{T}\kappa^{i}\beta \right\|}^{2}d_{ij}=const.\\ {\left\|\beta \right\|}_{1}=1,\beta_{m}\geq 0,m=1,2\ldots ,M\\ \kappa^{i}=\left(\begin{array}{ccc} K_{1}\left(1,i\right) & \cdots & K_{M}\left(1,i\right)\\ \vdots & \ddots & \vdots \\ K_{1}\left(N,i\right) & \cdots & K_{M}\left(N,i\right) \end{array}\right)\in R^{N\times M} \end{array}$$ (3)

where $\alpha ={\left[\alpha_{1}\ldots \alpha_{N}\right]}^{T}\in R^{N}$ is a projection vector, and $\beta ={\left[\beta_{1}\ldots \beta_{M}\right]}^{T}\in R^{M}$ is the kernel weight vector. A projection matrix $A=\left[\alpha_{1}\cdots \alpha_{p}\right]$ can be optimized for the case of the projection into more than one dimension. Then, ***A*** and ***β*** were optimized simultaneously using the coordinate descent algorithm. Specifically, the iterative optimization of A and β is performed alternately until reaching convergence or a maximum number of iterations. If ***A*** is optimized first, then we set the initial values for β as equal weights for all kernel matrices. If starting with the optimization of β , then we initialize ${AA}^{T}$ to the identity matrix *I.*

After mapping the similarities between each sample and its nearest neighbors to a low-dimensional space, *k*-means was used for clustering, and the optimal subtyping number was selected based on the silhouette coefficient (Rousseeuw et al., 1987).

#### 2.3.2 Evaluation of the biological differences between different HCC subtypes

We carried out survival analysis to explore whether the subtyping results correlated with patient survival outcomes and evaluate the clinical significance on survival rate of the identified subgroups. The Kaplan-Meier survival curve provides an intuitive measure of the survival risk for different subtypes, followed by the log-rank test to examine the difference of survival curves. Cox regression analysis was subsequently conducted on the HCC subtypes. A prognostic model based on the selected data was established after controlling for clinically relevant covariates.

Differentially expressed mRNAs (DEmRNAs), miRNAs (DEmiRNAs) and differentially methylated genes (DMGs) between subtypes were further explored. Specifically, the DEmRNAs satisfying the log2 fold change (FC)>1 & $P_{adj}<0.001$ and DEmiRNAs satisfying the log2 fold change (FC)>1 & $P_{adj}<0.01$ were further analyzed, using the DESeq2 R package (Love et al., 2014). The target DEmiRNA genes were then predicted using the miRTarBase (Chou et al., 2018) database. DMGs were selected using the Limma R package (Ritchie et al., 2015), following the criteria of $P_{adj}<0.001$ and |t|>2 . Finally, the comprehensive analysis of DEmRNAs, DEmiRNAs and DMGs was performed to obtain genes differentially expressed in different omics data. Then, to explore the relevant biological function categories and signaling pathways of these genes, Gene Ontology (GO) (Ashburner et al., 2000) and Kyoto Encyclopedia of Genes and Genomes (KEGG) (Kanehisa et al., 2012) enrichment analysis were conducted through the online KOBAS tool (Xie et al., 2011). The cutoff criterion is set to $P_{adj}<0.05$ .

#### 2.3.3 Biological pathway activity and immune cell infiltration analysis

To explore the biological changes that lead to survival differences between subtypes, pathway activity analysis was performed using PROGENy (Schubert et al., 2018). Non-parametric tests were used to identify biological pathways that were activated differently between subtypes with the threshold set as $P_{adj}<0.01$ . We also conducted the immune cell infiltration analysis to obtain significantly differential immune-infiltrating cells between different subtypes. Based on the immune infiltration data provided by Tumor Immune Estimation Resource (TIMER2.0) (Li et al., 2016; Li et al., 2020), the immune-infiltrating cell abundance of 287 patients with HCC were obtained. The Microenvironment Cell Populations-counter (MCP-counter) (Becht et al., 2016) algorithm was used to estimate tumor cell components. Then, significantly differential immune-infiltrating cells between subtypes were selected using non-parametric tests with the threshold set as $P_{adj}<0.01$ .

#### 2.3.4 Co-expression network construction and core module identification

Gene modules that affect the prognosis of HCC patients were identified with the weighted gene co-expression network analysis (WGCNA). In this work, the top 5,000 genes were selected according to the median absolute deviation to construct an mRNA co-expression network using WGCNA R package (Langfelder and Horvath, 2008). The brief implementation was as follows: setting the power of β as 6 (*R* ^2^ = 0.86), the gene co-expression correlation matrix was transformed into an adjacency matrix and then into a topological overlap matrix (TOM). A dynamic shear tree algorithm was applied to identify gene modules and further incorporated related modules following a height cutoff of 0.25. Finally, by associating module eigengene (ME) with clinical features, core modules that are related to patient outcomes were selected for subsequent analysis.

#### 2.3.5 Hub gene identification and prognostic evaluation

The candidate genes were defined as genes correlated with the ME and clinical traits. The ME is the most important component of a gene module and represents the gene module expression profile. The module membership (MM) of a gene represents the correlation of its gene expression profile with a specific ME. Candidate genes were defined as those correlated with the ME (cor. MM> 0.85) and clinical traits (cor. gene Trait Significance >0.30). The Maximal Clique Centrality (MCC) algorithm was then used to obtain highly connected genes from candidate genes using the cytoHubba plugin in Cytoscape software (v3.7.2) (Ceccarelli et al., 2020). The top 15 highly correlated genes were used as hub genes for further analysis.

We evaluated the prognostic value of hub genes by dividing patients into two groups according to the median value of hub gene expression. Patients equal or above the median value were categorized as the high-level group and those below the median were categorized as the low-level group. The statistical significance of survival outcomes in the two groups was assessed by survival analysis, screening for genes associated with prognosis (*p*-value < 0.05).

## 3 Results

### 3.1 Identification of HCC subtypes

**FIGURE 1:**
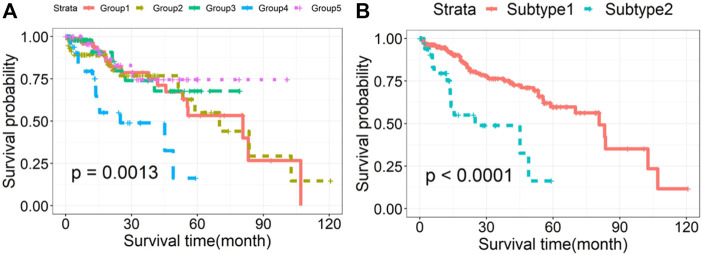
Kaplan-Meier survival curves of HCC subtypes identified by rMKL-LPP method. **(A)** The survival curves drawn based on the initial subtypes in HCC and **(B)** the survival curves of the regrouped Subtype 1 and Subtype 2.

The survival curves of HCC subtypes identified by rMKL-LPP method were given in Figure 1A. The survival probability between groups 1, 2, 4 and 5 was not statistically significant. Thus, we combined patients in the four groups into one group named as Subtype1, while named the group 3 as Subtype2. Thirty-nine patients (13.6%) in Subtype2 had 3-years mortality rate of 51%; while those in the Subtype1 had 3-years mortality rate of 23.5%. The basic characteristics of the two subtypes are shown in Table 1, and the survival curves are presented in Figure 1B. It can be seen that the two subtypes differed significantly in survival outcomes. Compared to Subtype1, Subtype2 had a significantly lower survival probability (*p* = 4.14E-05).

**TABLE 1:** Clinical characteristics of HCC subtypes.

Item	Subtype 1	Subtype 2
Cases, *n* (%)	248 (86.4)	39 (13.6)
Age, years	58.57 ± 13.00	59.15 ± 11.78
Female, *n* (%)	80 (32.3)	12(30.8)
Pathologic stage, *n* (%)		
Stage I	133 (53.6)	16 (41.0)
Stage II	59 (23.8)	13 (33.3)
Stage III	52 (21.0)	10 (25.7)
Stage IV	4 (1.6)	0
Death event, *n* (%)	48 (19.4)	14 (35.9)

To study the prognosis of different subtypes, the Cox regression model was constructed after controlling for age, gender and pathologic stage. The regression results are presented in Table 2. Patients in Subtype2 were 3.369 times higher in risk of death than Subtype1. Therefore, Subtype 1 was named the low-risk group and Subtype 2 was named the high-risk group. The only significant covariate is pathologic stage (*p* = 0.003).

**TABLE 2:** Results of Cox regression analysis in 287 patients with HCC.

Item	Coefficient (SE)	Wald Z	P	HR (95% CI)
Subtypes^a^	1.214(0.323)	3.756	<0.001	3.369 (1.787,6.349)
Age	0.005(0.011)	0.413	0.680	1.005 (0.983,1.026)
Gender	-0.191(0.282)	−0.678	0.498	0.826 (0.475,1.436)
Pathologic stage				
Stage II	0.010(0.348)	0.027	0.978	1.010 (0.511,1.996)
Stage III	0.285(0.312)	0.912	0.361	1.330(0.721,2.451)
Stage IV^a^	1.907(0.638)	2.990	0.003	6.730 (1.929,23.483)

a Shows the statistical significance at the α = 0.05 level.

### 3.2 Biological differences between the two subtypes

**FIGURE 2:**
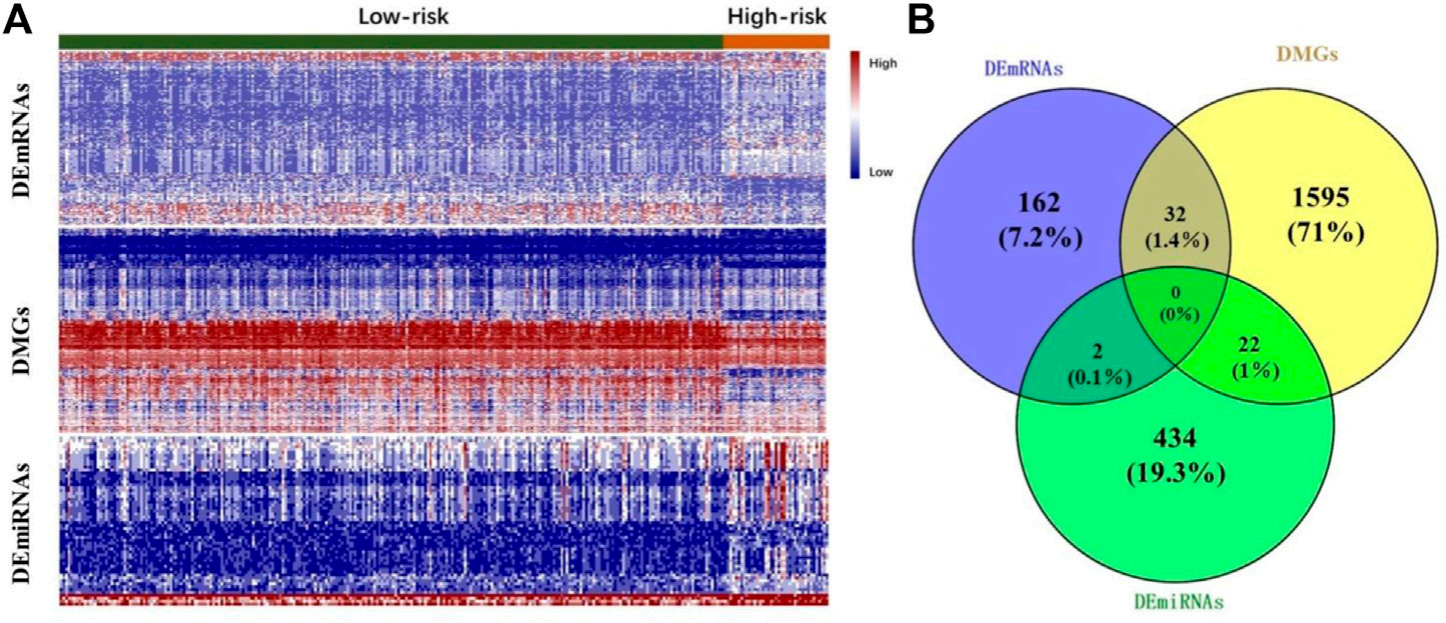
**(A)** Heatmap of DEmRNAs, DMGs and DEmiRNAs between the two subtypes. Each column corresponds to a patient and each row indicates an individual feature. The relatively high and low expression of genes are shown in red and green color respectively. **(B)** Venn diagram of differentially expressed gene analysis results in different omics data.

Focusing on the two subtypes, differential expression analysis was conducted for each omics data type. Based on the pre-set threshold (see Section 2.3.2), a total of 196 DEmRNAs were selected, where 132 were upregulated and 64 were downregulated; 58 DEmiRNAs were selected, among which 56 were upregulated and 2 were downregulated. A total of 2,151 DMGs were also identified, where 1,254 were hypermethylated and 897 were hypomethylated. Figure 2A shows a heatmap of differentially expressed profiles of different omics data between the two subtypes. The heatmap showed that the expression profiles among the three omics data types are different between high- and low-risk groups of HCC. A total of 458 genes targeted by 58 DEmiRNAs were predicted. Then we performed the comprehensive analysis of differentially expressed genes (DEGs) in different omics data. As shown in Figure 2B, 32 genes were observed to be differentially expressed in mRNA, along with abnormal methylation; 22 genes were abnormally methylated and differentially expressed in miRNA, and 2 genes were differentially expressed in mRNA and their corresponding miRNA.

**FIGURE 3:**
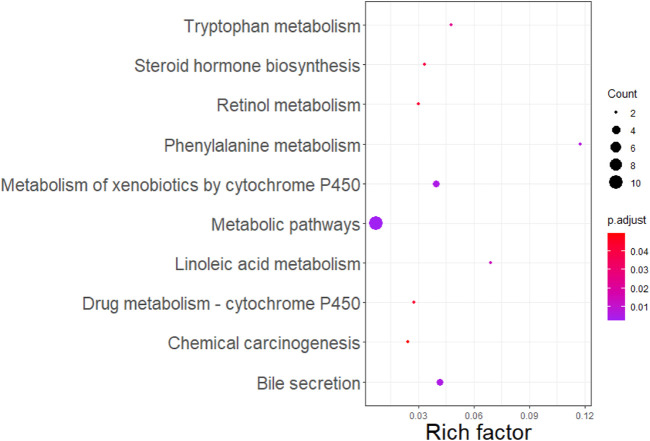
KEGG enrichment analysis for 56 genes selected.

**FIGURE 4:**
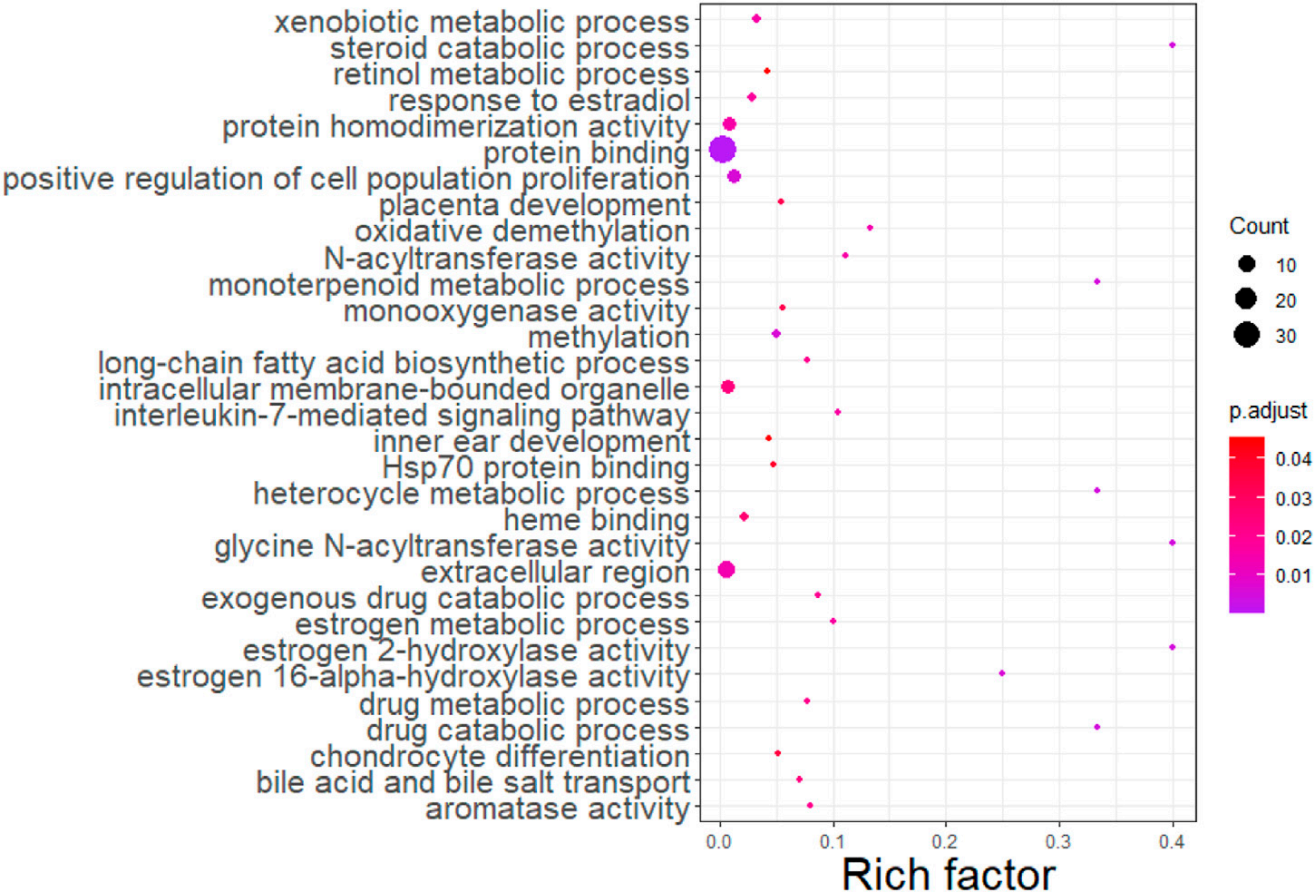
GO enrichment analysis for 56 genes selected.

We further merged these 56 genes into a core set for KEGG pathway and GO enrichment analysis. KEGG analysis indicated that these genes were enriched in 10 pathways (see Figure 3). In addition, these genes were mainly enriched in 31 GO terms (see Figure 4).

### 3.3 Pathway activity and immune cell infiltration analysis

**FIGURE 5:**
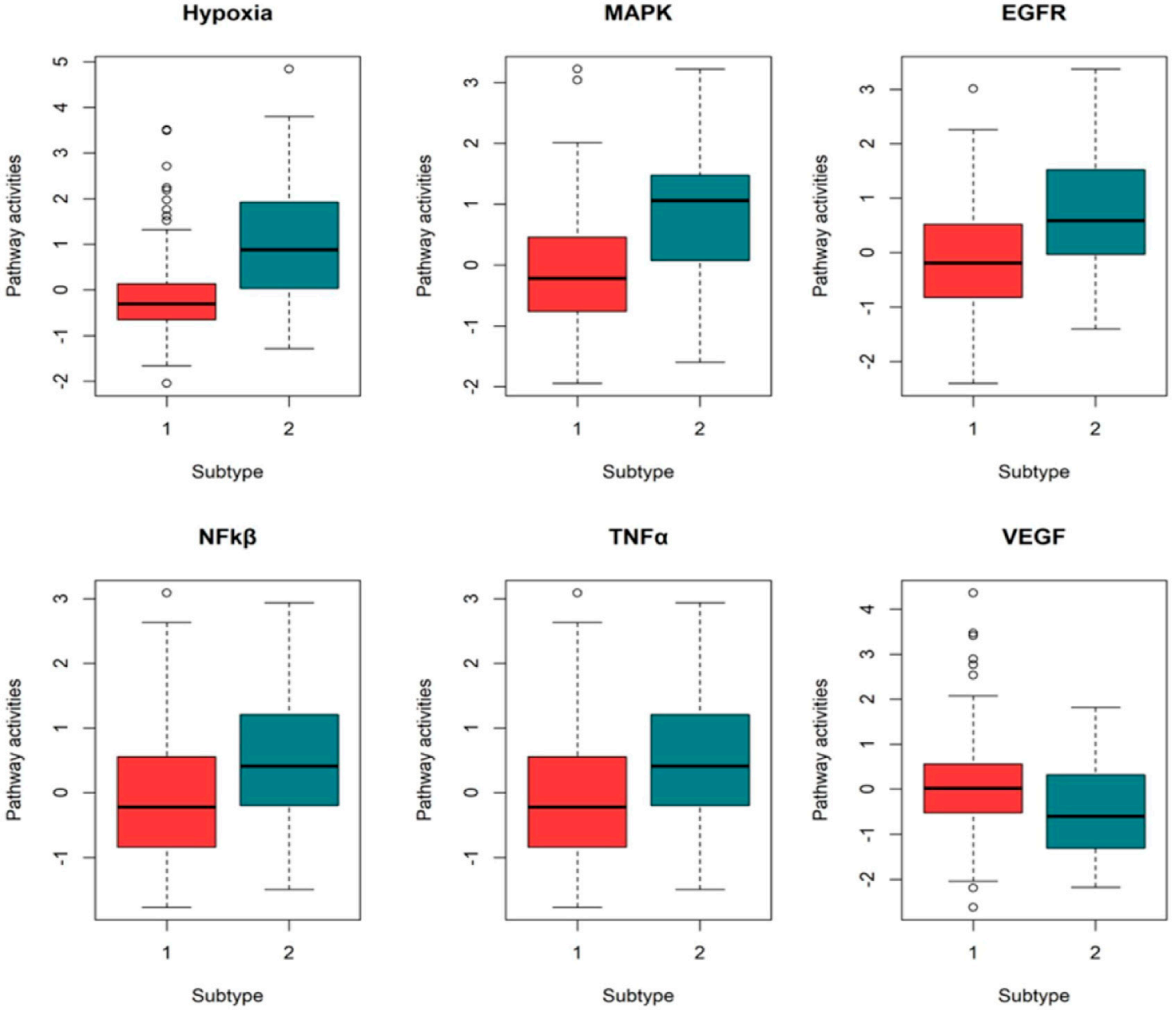
Boxplots showing the pathway activity for six pathways.

We performed pathway activity and immune cell infiltration analysis to further explore the biological and clinical meaning of the two subtypes. As shown in Figure 5, the activities of 6 pathways between two subtypes were significantly different. Specifically, the activities of Hypoxia, MAPK, EGFR, NF-kβ, and TNFα pathways was found to be significantly higher in the high-risk group than in the low-risk group; while VEGF pathway activity showed higher in the low-risk group.

**FIGURE 6:**
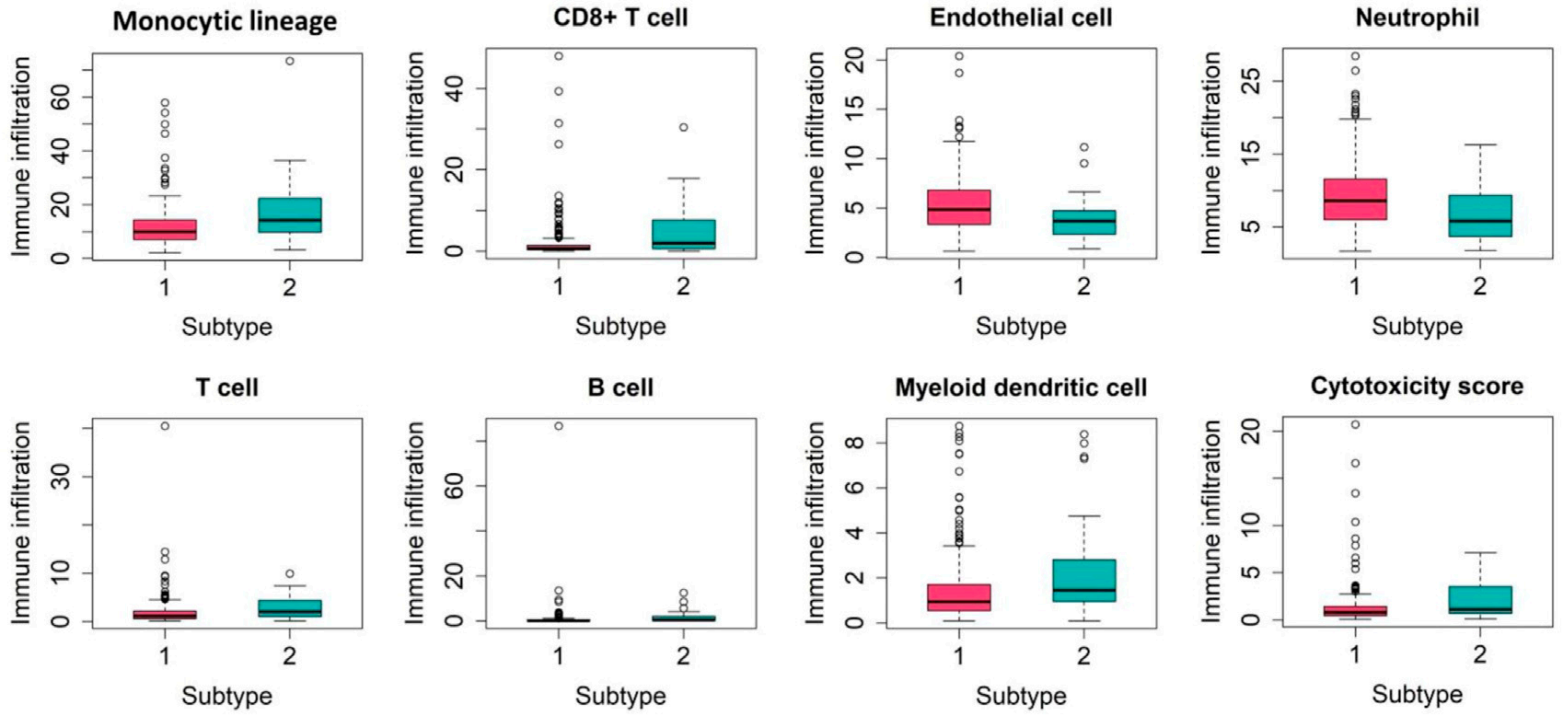
Boxplots showing the abundance of nine immune cells between the two subtypes.

Figure 6 revealed that the abundance of 9 immune cells differed significantly between two subtypes. The abundance of monocytic lineage, CD8+T cell, T cell, myeloid dendritic cell, and cytotoxicity score was found to be significantly higher in the high-risk group than in the low-risk group. Tumor-infiltrating immune cells are closely related to clinical outcomes of patients in many types of tumors and are likely to serve as target spots in cancer-targeting drug delivery systems. This provides ideas for a targeted therapeutic strategy of HCC.

### 3.4 Core module and hub gene identification

**FIGURE 7:**
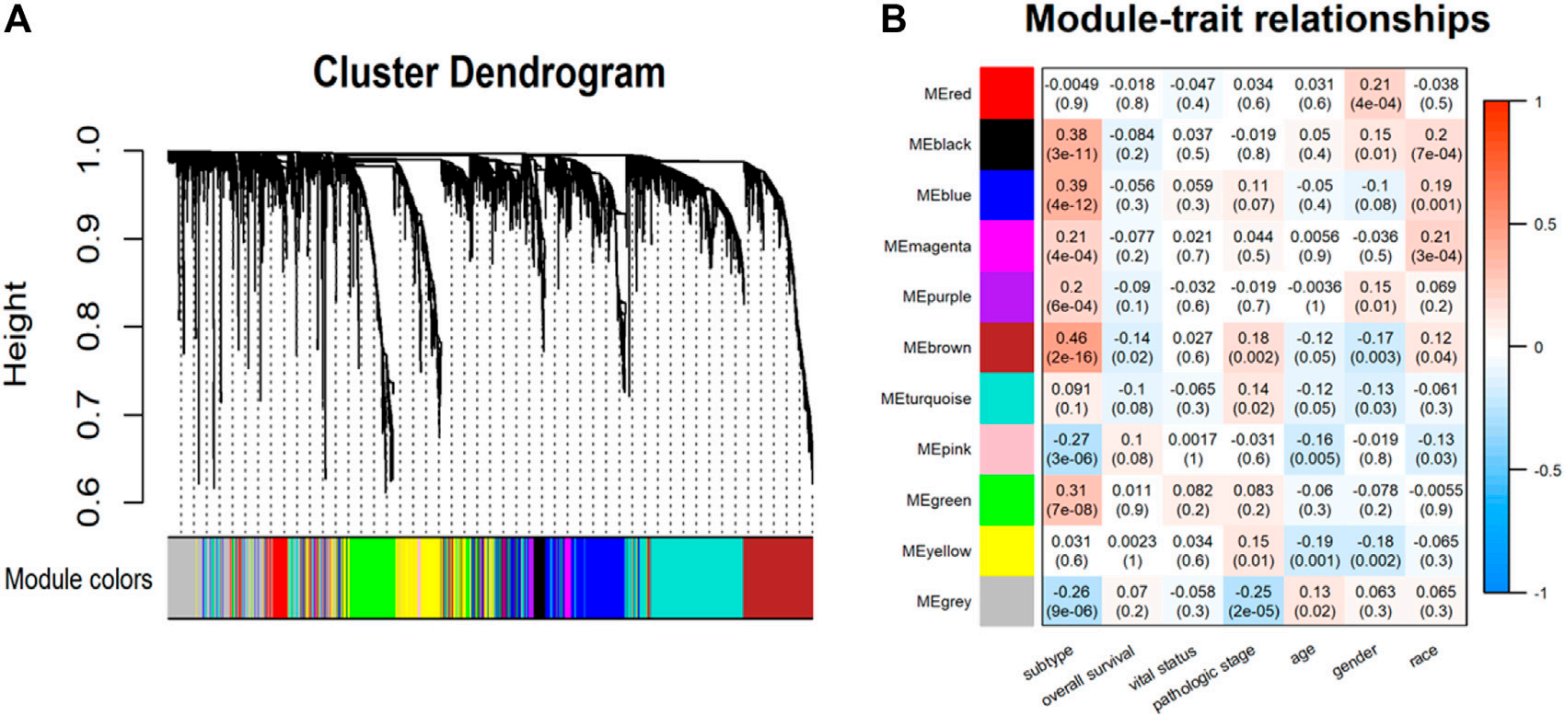
**(A)** Hierarchical clustering dendrogram of identified co-expression modules. **(B)** Heatmaps of the correlation between modules and clinical traits. Each row represents a module and each column represents a clinical feature. Each cell consists of the correlation and *p*-value (in parenthesis).

**FIGURE 8:**
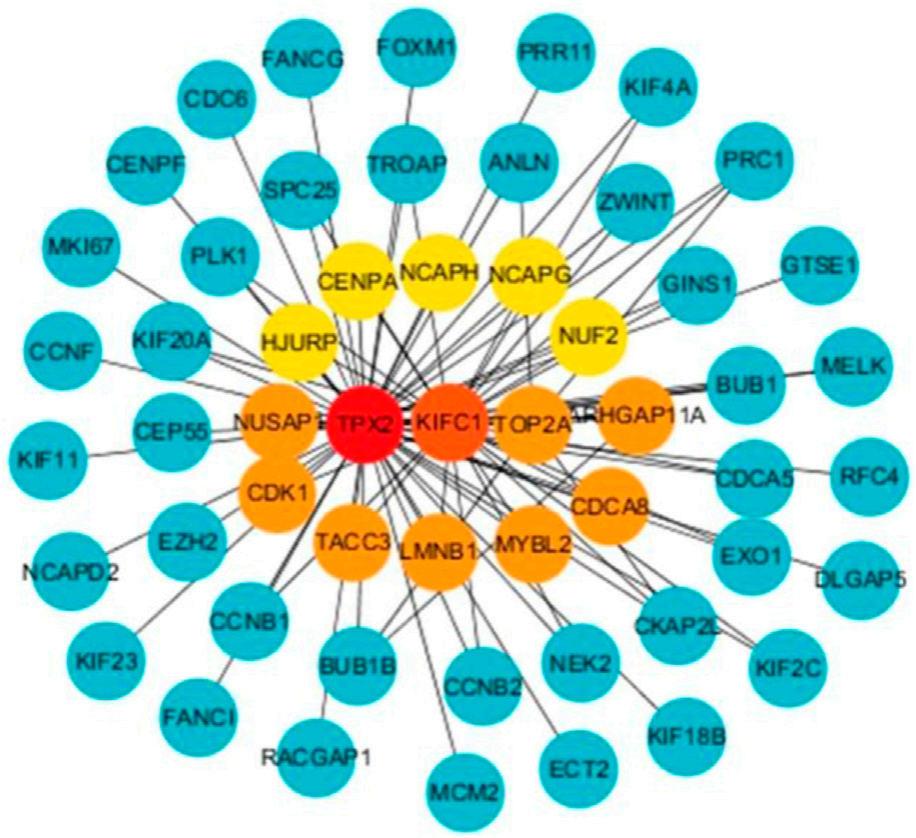
Network diagram of interaction between hub genes and candidate genes.

In the WGCNA analysis, 11 co-expression modules were identified (Figure 7A). Among them, the brown module that is significantly related to subtype (*r* = 0.46, *p* < 0.0001) was selected for subsequent analysis (Figure 7B). Then, 59 candidate genes were screened from the brown module that included a total of 758 genes based on the preset criteria. The CytoHubba plugin in Cytoscape software was employed to measure the MCC score of candidate genes to identify hub genes. Finally, the top 15 genes were selected as hub genes for further analysis by sorting the MCC score. They were *TPX2*, *KIFC1*, *MYBL2*, *TOP2A*, *NUSAP1*, *ARHGAP11A*, *LMNB1*, *CDK1*, *CDCA8*, *TACC3*, *NUF2*, *NCAPG*, *HJURP*, *NCAPH*, and *CENPA*. The interaction between hub genes and candidate genes was visualized using Cytoscape software. Shown in Figure 8, each candidate gene is less connected to all other candidate genes and more connected to hub genes.

### 3.5 Evaluation of prognostic value of hub genes

**FIGURE 9:**
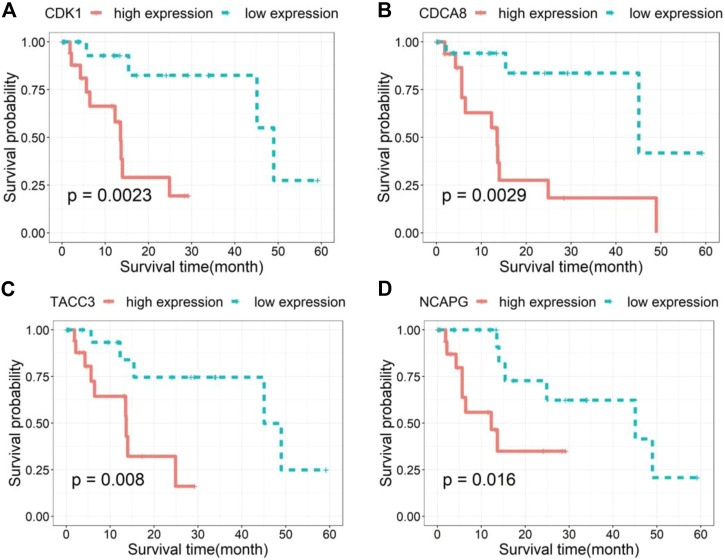
Survival curves **(A–D)** of the four hub genes sorted in ascending order of *p*-value.

We further investigated the links between hub genes and the prognosis of patients in Subtype2 separately using the Kaplan-Meier method. Four of the 15 hub genes (*CDK1*, *CDCA8*, *TACC3*, and *NCAPG*) significantly correlated with prognosis (*p* < 0.05). As shown in Figure 9, these genes with high expression were accompanied by a poor prognosis in Subtype2 patients, indicating the role of these genes in the high-risk group.

## 4 Discussion

In this study, we applied rMKL-LPP method to integrate three omics data types (mRNA expression, miRNA expression and DNA methylation) with 287 patients for HCC. Coupling with the survival analysis, these patients were further classified into two subtypes which show significant association with overall survival. The high-risk group had a higher 3-years mortality rate of 51.0% while the low-risk group had a 3-years mortality rate of 23.5%. Furthermore, the death risk of HCC patients in the high-risk group was found to be 3.37 times higher than that in the low-risk group. Focusing on the two subtypes, potential diagnostic biomarkers (genes or signaling pathways) were identified through bioinformatics analysis. The present results provided an important reference for future precision treatment of HCC patients.

We performed the differential expression analysis and pathway activity analysis to reveal the biological changes that contribute to survival differences between two subtypes. Abnormal expression of DNA methylation and miRNA can occur at all stages of HCC development and play a cancer-promoting or carcinostatic role through several mechanisms (Zheng et al., 2019). When analyzing the interactive relationship among DEmiRNAs, DEmRNAs and DMGs, 56 DEGs were selected. Some of the DEGs may serve as potential biomarkers of HCC. For example, *CLEC4M* and *CYP2C8* have been reported as potential prognostic biomarkers in patients with HCC (Li et al., 2019; Luo et al., 2020). Both *PPOX* and *HMBS* play key roles as tumor suppressors in the hepatocarcinogenesis (Schneider-Yin et al., 2015). *APLN* can be used as an independent prognostic factor for HCC (Chen et al., 2019). *ANXA2* and *C8orf33* have been reported as key genes to distinguish poorly differentiated HCC and well-differentiated HCC (Shao et al., 2017). Different pathways play different roles in multiple biochemical and pathological mechanisms of hepatocarcinogenesis. The activity of the six pathways: Hypoxia, MAPK, EGFR, NF-kβ, TNFα and VEGF pathway varied significantly across subtypes. Pathways such as the TNFα and NF-kβ were found to have a procardiogenic effect on the liver (Pikarsky et al., 2004; Ramakrishna et al., 2013). Recently, (Liu et al., 2020), found that combination therapy, involving anti-VEGF and ICBs, could potentially benefit patients with HCC. This suggests that pathway-blocking therapy can provide new opportunities for precise treatment of HCC.

We also performed WGCNA analysis to identify gene modules and genes affecting the prognosis of HCC patients. The results demonstrated that the brown module was most strongly associated with prognosis. This indicated that the critical genes in the brown module may serve as potential biomarkers affecting the progression of HCC. Further analysis found that 4 out of 15 hub genes were closely correlated with the prognosis of Subtype2 patients. These 4 genes have also been reported in the occurrence and development of HCC. *NCAPG* plays a substantial role in genetic factors that modulate fetal growth (Eberlein et al., 2009) and is associated with vascular invasion in HCC (Guo and Zhu, 2021). Some studies have reported that *NCAPG* dysregulation is associated with cancers, including gliomas and melanomas (Ryu et al., 2007). *CDCA8*, a key component of the chromosome passenger complex, regulates cell dynamic localization during mitosis (Cui et al., 2021). High expression of *CDCA8* may lead to poor prognosis in patients with lung and gastric cancer. *CDK1*, a catalytic subunit of the highly conserved protein kinase complex, may serve as a potential target for lycorine against HCC (Yin et al., 2021). *TACC3* is responsible for cell mitosis and transcriptional functions. Its high expression is positively associated with poor overall survival (Zhou et al., 2015).

In summary, the present research integrated HCC multi-omics data and effectively identified subtypes using rMKL-LPP method, which provides novel strategies and ideas for the subtyping study of HCC. In addition, the selected potential pathogenic genes, pathways and tumor-infiltrating immune cells can be used as references to control related gene expression or interfere with their target signal transduction pathways to provide potential opportunities for the treatment of HCC. For future research, the limitations of the present study must be acknowledged. More adequate experiments are needed to confirm the role of potential biomarkers and further validation of the HCC subtypes identified in this study is needed.

## Data Availability

Publicly available datasets were analyzed in this study. This data can be found here: https://www.cancer.gov/about-nci/organization/ccg/research/structural-genomics/tcga.
